# Involvement of the CB_2_ cannabinoid receptor in cell growth inhibition and G0/G1 cell cycle arrest via the cannabinoid agonist WIN 55,212–2 in renal cell carcinoma

**DOI:** 10.1186/s12885-018-4496-1

**Published:** 2018-05-23

**Authors:** Mohammed I. Khan, Anna A. Sobocińska, Klaudia K. Brodaczewska, Katarzyna Zielniok, Malgorzata Gajewska, Claudine Kieda, Anna M. Czarnecka, Cezary Szczylik

**Affiliations:** 1 Molecular Oncology Laboratory, Department of Oncology, Military Institute of Medicine, ul. Szaserów 128, 04-141 Warsaw, Poland; 2 Faculty of Biology, Warsaw University, ul. Miecznikowa 1, 02-096 Warsaw, Poland; 3 Department of Physiological Sciences, Warsaw University of Life Sciences-SGGW, Nowoursynowska 159, 02-776 Warsaw, Poland; 4 Department of Otolaryngology - Head & Neck Surgery, Western University, London, ON N6A 3K7 Canada; 5 Warsaw Medical University, Żwirki i Wigury 61, 02-091 Warsaw, Poland

**Keywords:** Renal cell carcinoma, Endocannabinoid system (ECS), CB_1_ and CB_2_ receptors, JWH-133, WIN 55,212–2 mesylate

## Abstract

**Background:**

The anti-tumor properties of cannabinoids have been investigated in many in vitro and in vivo studies. Many of these anti-tumor effects are mediated via cannabinoid receptor types 1 and 2 (CB_1_ and CB_2_), comprising the endocannabinoid system (ECS). In this study, we investigated the ECS based on *CB*_*1*_ and *CB*_*2*_ receptor gene and protein expression in renal cell carcinoma (RCC) cell lines. In view of their further use for potential treatments, we thus investigated the roles of CB_1_ and CB_2_ receptors in the anti-proliferative action and signal transduction triggered by synthetic cannabinoid agonists [such as JWH-133 and WIN 55,212–2 (WIN-55)] in RCC cell lines.

**Methods:**

Human RCC cell lines were used for this study. The *CB*_*1*_ and *CB*_*2*_ gene expression levels were analyzed using real-time PCR. Flow cytometric, immunocytochemical and western blot analyses were performed to confirm CB_1_ and CB_2_ receptor protein expression. The anti-proliferative effects of synthetic cannabinoids were investigated on cell viability assay. The CB_1_ and CB_2_ receptors were blocked pharmacologically with the antagonists SR141716A and AM-630, respectively, to investigate the effects of the agonists JWH-133 and WIN-55. Cell cycle, apoptosis and LDH-based cytotoxicity were analyzed on cannabinoid-treated RCC cells.

**Results:**

The *CB1* and *CB2* genes expression was shown by real-time PCR and flow cytometric and western blot analysis indicating a higher level of CB_2_ receptor as compared to CB_1_ in RCC cells. Immunocytochemical staining also confirmed the expression of the CB_1_ and CB_2_ proteins. We also found that the synthetic cannabinoid agonist WIN-55 exerted anti-proliferative and cytotoxic effects by inhibiting the growth of RCC cell lines, while the CB_2_ agonist JWH-133 did not. Pharmacologically blocking the CB1 and CB2 receptors with their respective antagonists SR141716A and AM-630, followed by the WIN-55 treatment of RCC cells allowed uncovering the involvement of CB2, which led to an arrest in the G0/G1 phase of the cell cycle and apoptosis.

**Conclusions:**

This study elucidated the involvement of CB_2_ in the in vitro inhibition of RCC cells, and future applications of CB_2_ agonists in the prevention and management of RCC are discussed.

## Background

Renal cell carcinoma (RCC) is the most common renal epithelial cancer in adults, accounting for more than 90% of all renal malignancies [1, 2]. The most important life-threatening factor in RCC is the metastatic dissemination of disease if RCC is not detected before the onset of metastasis. Approximately 30% of RCC patients are diagnosed with metastatic RCC, and 60% of these patients have a higher mortality rate due to the aggressiveness of metastatic RCC [3, 4].

RCC treatment is less effective because of the limited or lack of responsiveness to conventional therapies such as surgery and chemo/radiotherapies [5]. Targeted therapies are considered the standard care for the treatment of RCC and include tyrosine kinase inhibitors (TKIs) [6], monoclonal antibodies directed against vascular endothelial growth factor (VEGF) combined with interferon-alpha (IFNα) [7], mammalian target of rapamycin (mTOR) inhibitors [8] and, most recently, anti-programmed death-1 (PD-1) monoclonal antibody [9]. Despite all of the recent advancements in RCC diagnosis and treatment, the current therapies are unable to completely eliminate RCC cells, which persist after treatment. Controlling cancer growth and the development of chemo-preventive agents are the major goals in current basic research in oncology.

For many centuries, extracts from *Cannabis sativa* have been used for medicinal and recreational purposes. Cannabinoids, the active components of *Cannabis sativa*, are involved in a wide spectrum of physiological and pathological conditions, including inflammation, immunomodulation, analgesia and anti-tumor actions [10]. The primary active component of this plant is Δ^9^-tetrahydrocannabinol (THC), which was first explored in 1960 [11]. To date, approximately 66 unique compounds have been explored from *Cannabis sativa*, which were further classified into three categories: I) phytocannabinoids; II) endogenous cannabinoids; and III) synthetic cannabinoids [12]. Cannabinoids produce effects through the activation of two G-protein-coupled receptors, cannabinoid receptor type 1 (CB_1_) and cannabinoid receptor type 2 (CB_2_), which are responsible for the transduction of intercellular signals. The CB_1_ receptor is highly expressed in the brain and is related to the psychoactivity of cannabinoids. The CB_2_ receptor is unrelated to cannabinoid psychoactivity; therefore, selective CB_2_ activation may provide some of the therapeutic aspects of cannabinoids [13]. One of the most exciting research areas is the therapeutic application of cannabinoids in cancer and the development of these compounds as cancer treatments [12]. The anti-tumor properties of cannabinoids have been investigated in both in vitro and in vivo experiments, examining effects on multiple signaling pathways and biological processes that are involved in the development of the malignant phenotype [14]. The anti-tumor actions of cannabinoids include the induction of cell death, the inhibition of cell migration, metastasis and tumor cell proliferation, anti-angiogenic effects and the modulation of the immune response, suggesting the potential use of cannabinoids in the treatment of various cancers of the breast, prostate, lungs, pancreas, and bladder as well as gliomas [12, 15–20].

The CB_1_ and CB_2_ receptors comprise the endocannabinoid system (ECS) within a cell. There is growing evidence suggesting that the ECS and synthetic cannabinoids modulate the enzymes and nuclear factors involved in cancer cell homeostasis, growth, migration and tumor angiogenesis [10, 12, 14, 17, 21]. The activation of CB_1_ or CB_2_ within the ECS leads to the activation of corresponding signaling pathways involved in tumor processes, including the PI3K/Akt pathway, the regulation of adenylyl cyclase, the cyclic AMP-protein kinase-A (cAMP-dependent PKA) pathway, ERK (extracellular signal-regulated kinase) and MAPK (mitogen-activated protein kinase) [12, 14]. Additionally, the ECS is an attractive potential target for cancer therapy because of the unique capability of the ECS to select cancer cells from among non-tumor cells.

**Fig. 1:**
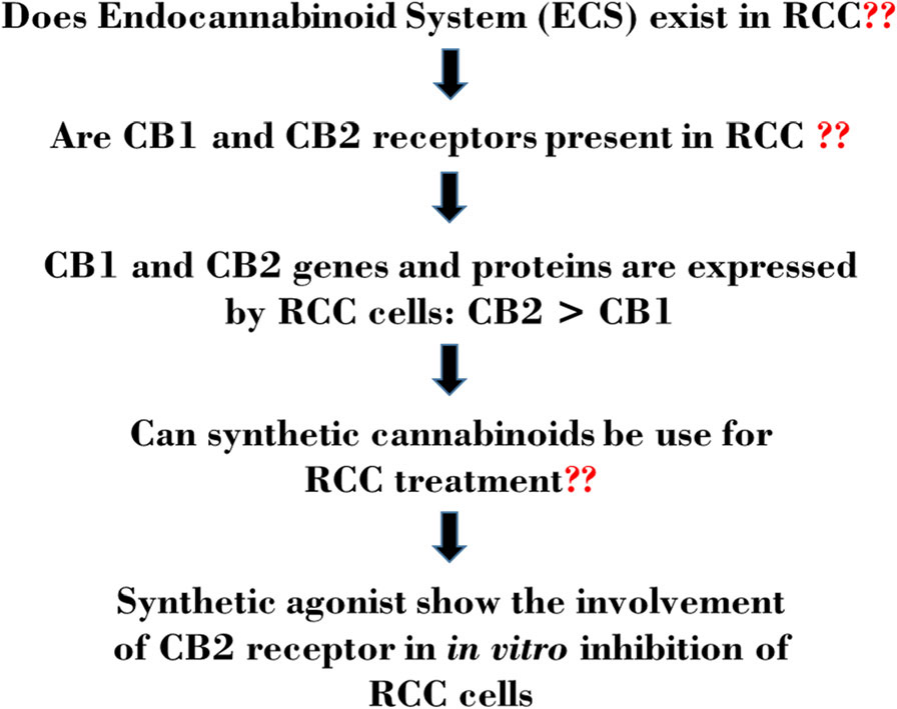
Workflow of ECS study in RCC cells

The purpose of this study was to investigate the ECS of RCC cells based on CB_1_ and CB_2_ receptor expression. In this study, we analyzed the gene and protein expression of the CB_1_ and CB_2_ receptors in RCC cell lines. We used the CB_1_ and CB_2_ receptor agonists JWH-133 and WIN 55,212–2 (WIN-55) in assessing the anti-proliferative actions against RCC cells. The CB_1_ and CB_2_ receptors were blocked pharmacologically with antagonists specific for the CB_1_ and CB_2_ receptors, SR141716A and AM-630, respectively, to reveal the roles played by these receptors in signal transduction. Figure 1 shows the workflow of research carry out in this study.

## Methods

### RCC cell culture

Human primary RCC cell lines (786-O, SMKT-R2, SMKT-R3, Caki-2, RCC-6, 769-P), metastatic cell lines (Caki-1 and ACHN) and a healthy human kidney epithelial cell line (ASE-5063) were used for cell cultures and experiments. All of the cell lines were obtained and cultured as previously described [22]. The cells were expanded in T-75 flasks, T-25 flasks and 96-, 24-, and 6-well plates (Orange Scientific, Braine-l’Alleud, Belgium), as well as 4-chamber slides (ThermoFisher Scientific, Massachusetts, USA) depending on the experiments. Confluent cell monolayers were harvested with Accutase™ Cell Detachment Solution (BD Biosciences, California, USA).

### Reagents

JWH-133, WIN-55 mesylate, AM-630 and SR141716A were purchased from Tocris Bioscience (Bristol, United Kingdom). Anti-CB_1_, anti-CB_2_ and anti-GAPDH antibodies were purchased from Abcam (Cambridge, United Kingdom). DAPI, Alexa Fluor® 546 secondary goat anti-rabbit antibody and Pierce™ LDH Cytotoxicity Assay Kit were purchased from ThermoFisher Scientific (Massachusetts, USA). The Muse™ PI3K/MAPK Dual Pathway Activation Kit (MCH200108) was purchased from Merck EMD Millipore (Massachusetts, USA) to assess the activation of PI3K and MAPK signaling pathways. The Alamar Blue® cell viability reagent was purchased from Invitrogen (California, USA) for the cell proliferation assay.

### Reverse transcription and real-time PCR

Total RNA from RCC cell lines and the healthy human kidney epithelial cell line ASE-5063 was isolated using Total RNA Mini Plus (A&A Biotechnology, Gdynia, Poland) as previously described [3]. The RNA quality and concentrations were determined by measuring the absorbance at 230 nm, 260 nm, and 280 nm using the μDrop plate from a Multiskan™ GO microplate spectrophotometer (ThermoFisher Scientific, Massachusetts, USA). A Maxima H Minus First Strand cDNA Synthesis Kit with dsDNase (ThermoFisher Scientific, Massachusetts, USA) was used for the cDNA synthesis as described in the protocol. Real-time PCR was performed using a LightCycler® Nano Instrument (Roche, Basel, Switzerland). CB_1_ and CB_2_ genes were amplified using primers described previously [23]. Each gene was analyzed in separate PCR tubes (in triplicate) using the FastStart Essential DNA Green Master Mix kit from Roche according to the manufacturer’s protocol (Basel, Switzerland). The mRNA expression levels of the *CB*_*1*_ and *CB*_*2*_ receptor genes were compared with that of the *PPIA* (123 bp) gene as an endogenous control. As a negative control, no cDNA was added to the PCR tubes containing the FastStart Essential DNA Green Master Mix to determine whether all of the reagents were free of the target sequence. The total RNA from ASE-5063 cells was used as a positive control for the *CB*_*1*_ and *CB*_*2*_ genes. The data were obtained using LightCycler® Nano software 1.0 (Roche, Basel, Switzerland). The relative mRNA expression levels were then normalized using the mRNA level of the reference gene (*PPIA*) as the endogenous control in each sample. The mRNA data were analyzed using the Comparative Ct method [24].

### Flow cytometry

Cells were cultured in T-25 flasks as described above for flow cytometric analysis. The cells were harvested using Accutase, and the cell number was determined. The cells were centrifuged and re-suspended in Fc receptor (FcR) for 15 min at 4 °C. Anti-CB_1_ and anti-CB_2_ primary antibodies were then added to 10^4^ cells at a dilution of 1:1000 (for the CB_1_ and CB_2_ antibodies), and the cells were incubated for 20 min at 4 °C. The cells were then washed and centrifuged 3 times before adding AlexaFluor® 546 secondary goat anti-rabbit antibody (1:400) and incubating for 20 min in the dark (4 °C). The cells were washed twice with cold phosphate-buffered saline (PBS) before data acquisition using a FACSCalibur (BD Biosciences, California, USA). The flow cytometric data analysis and generation of dot plots and histograms were performed using FCS Express 5.1 (DeNovo Software, California, USA).

### Immunocytochemistry (ICC)

RCC cells were cultured in 4-chamber slides as described above. At approximately 80% confluence, the monolayer cell culture was rinsed briefly in PBS. Next, the cells were covered in 4% paraformaldehyde (PFA) for 10 min at room temperature. The PFA was removed by washing with PBS (3 times) and goat serum (10%) in PBS was used for blocking for 1 h at room temperature. The cells were incubated separately with diluted primary antibodies against CB_1_ (1:1000) and CB_2_ (1:1000) at 4 °C for 4 h. The cells were washed three times with PBS and then incubated with Alexa Fluor® 546 secondary goat anti-rabbit antibody (1:400) for 2 h at room temperature away from light. Again, the cells were rinsed 3 times with PBS, followed by incubation with DAPI (1:5000) (ThermoFisher Scientific, Massachusetts, USA) for 10 min. For the control, the cells were incubated only with secondary antibody. The slides were washed with PBS and covered with coverslips using CoverGrip Sealant (Biotium, California, USA), and images were captured using an Olympus CKX41 fluorescence microscope.

### Western blot analysis

Western blot assays were performed to analyze the protein expression of the CB_1_ and CB_2_ receptors in the RCC cell lines. Forty micrograms of total protein was solubilized in Laemmli sample buffer and resolved by electrophoresis in 12% Precise Tris-Glycine Gels (ThermoFisher Scientific, Massachusetts, USA). Next, proteins were transferred to polyvinylidene difluoride membranes. The membrane blots were blocked for 2 h in skimmed milk and were incubated overnight with primary antibodies against CB_1_ (1:500), CB_2_ (1:500), and GAPDH (1:2000). Finally, the membrane blots were washed and incubated for 1 h with the secondary antibody IRDye® 800 CW goat anti-rabbit IgG (1:5000). Immunoreactive bands were visualized using the Odyssey infrared imaging system (LI-COR Biosciences, Nebraska, USA). Quantification of the integrated optical density (IOD) of the bands was performed using analysis software as previously described [25]. For the quantitative analysis, the relative IOD of both the CB_1_ and CB_2_ target proteins was normalized to the IOD of GAPDH.

### Alamar blue® cell viability and LDH-based cytotoxicity assay

RCC cells were seeded at a density of 2000 cells per well in 96-well plates and were cultured in RPMI-1640+ GlutaMAX™-I medium with 10% fetal bovine serum (FBS) under normoxic (20% O_2_) conditions at 37 °C with 5% CO_2_. After treatment with JWH-133, WIN-55 mesylate, AM-630 and SR141716A, the cell viability was analyzed using the Alamar Blue® cell viability assay as described previously [26]. LDH-based cytotoxicity assay was performed according to manufacturer protocol.

### Apoptosis and cell cycle analysis

Cell cycle analysis was performed using the Muse™ Cell analyzer (Millipore, Massachusetts, USA) following the manufacturer’s protocol. The analysis of apoptosis was performed by dual staining with Annexin V-FITC and propidium iodide (PI) using a FACSCalibur flow cytometer. To assess the cell cycle and analyze apoptosis induced by treatment of RCC cells with WIN-55, a total of 5000 cells were seeded in each well of a 6-well plate and expanded until the cells reached 70–80% confluency; the cells were then treated with increasing concentrations of WIN-55 as described above. Control cells were treated with only complete medium. After 48 h of incubation, the cells were harvested using an Accutase cell detachment solution and were stained with Annexin V-FITC and PI as previously described [27] for apoptosis analysis or were stained with the Muse™ cell cycle reagent according to the manufacturer’s protocol for cell cycle analysis.

### Sphere formation assay

In order to investigate the effect of WIN-55 treatment on RCC cells ability to form 3D spheres/colonies, cells were cultured and harvested as described above and washed twice with PBS to remove any FBS present in cell culture media. Cells were counted and seeded at density of 100 cells/well in ultra-low attachment 96 wells plates (TC plate, suspension, F, Sarstetd, Numbrecht, Germany) supplemented with sphere promoting media as described previously [28]. Later WIN-55 (0 μM (control), subtoxic concentration (10 μM) prepared in sphere promoting media) was added in wells with cells in the beginning (day 0), at the moment when cells started to form spheres (day 2–3) and at the end when spheres were formed (day 6–7). Scheme of experiment design were presented in Fig. 7 (a) (d) and (g). Spheres were counted and pictures were taken using Olympus CKX41 microscope for analysis.

### Analysis of PI3K/Akt and MAPK/ERK pathway activation

RCC cells (786-O and ACHN) were seeded at a density of 5000 cells per well in 6-well plates and were treated with increasing concentrations of WIN-55 as described above. The Muse™ PI3K/MAPK Dual Pathway Activation Kit (MCH200108) was used to evaluate the activation of the PI3K/Akt and MAPK/ERK signaling pathways simultaneously by WIN-55 treatment. The assay was performed according to the manufacturer user’s guide.

### Statistical analysis

The data were expressed as the means±standard deviation (SD) of at least three experiments. Statistical analysis and data fitting were performed and graphs were prepared using the StatSoft program STATISTICA 12 (Dell Statistica, Oklahoma, USA) and Microsoft’s Excel program 2013 (Washington, USA). The significance of differences was analyzed using the Student’s t test or an ANOVA. A *p* value < 0.05 was considered to indicate statistical significance.

## Results

### mRNA expression of *CB*_*1*_ and *CB*_*2*_ in RCC cells

**Fig. 2:**
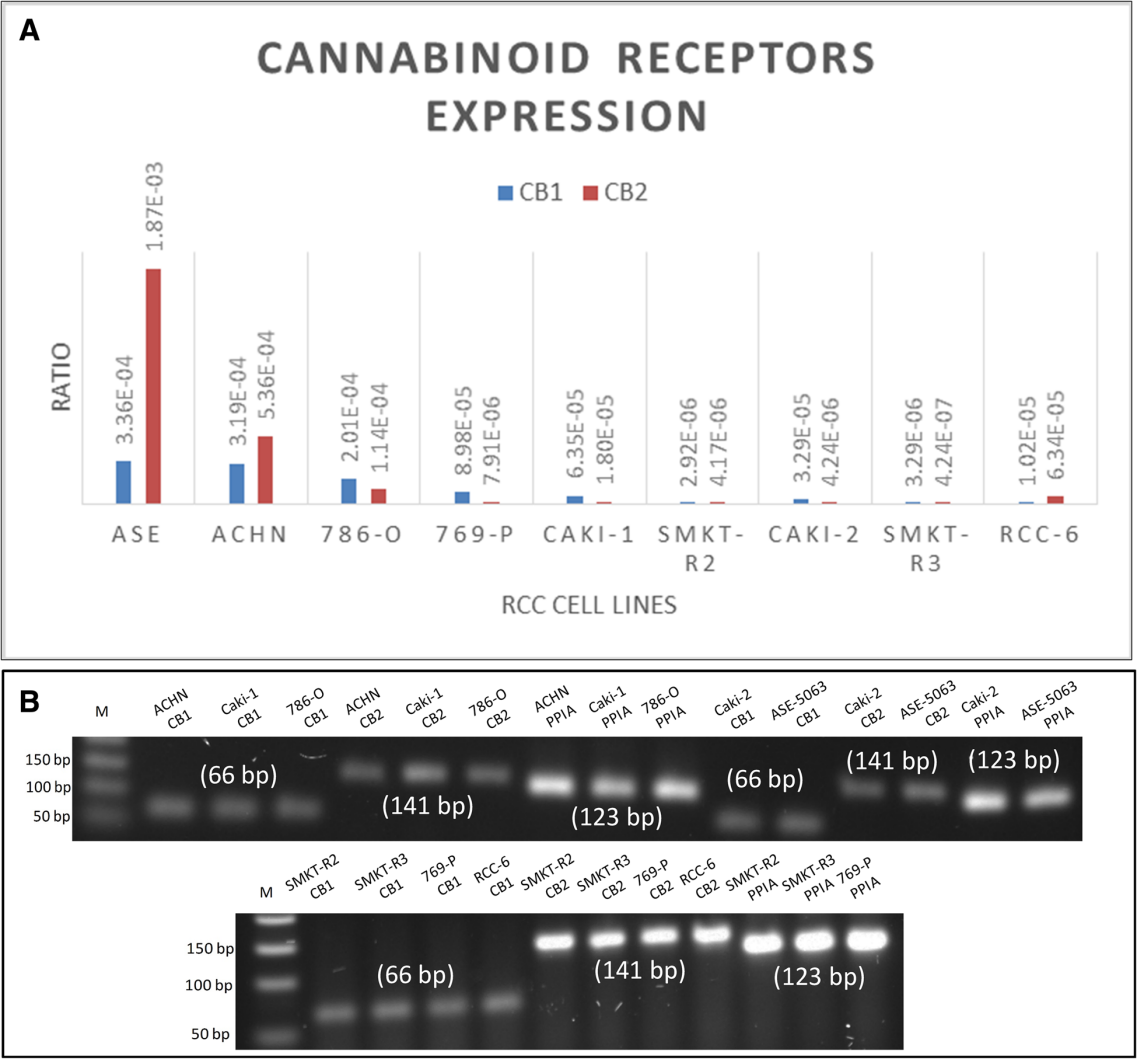
mRNA expression of the cannabinoid receptors *CB*_*1*_ and *CB*_*2*_ in different RCC cell lines. **a** The quantitative data indicate the expression of the *CB*_*1*_ and *CB*_*2*_ receptor genes in RCC cells. ASE-5063 (ASE) cells were used as a control for the *CB*_*1*_ and *CB*_*2*_ receptor genes. **b** Two agarose gels showing the presence of mRNA expression of *CB*_*1*_ (66 bp), *CB*_*2*_ (141 bp) and *PPIA* (123 bp) (endogenous control gene) in the RCC cell lines ACHN, Caki-1, 786-O, Caki-2, SMKT-R2, SMKT-R3, 769-P, and RCC-6, as well as in the healthy kidney cell line ASE-5063. M indicates the molecular marker

The primary goal of this experiment was to investigate the mRNA expression of the cannabinoid receptors *CB*_*1*_ and *CB*_*2*_ in RCC cells. Our real-time PCR results revealed the expression of *CB*_*1*_ and *CB*_*2*_ genes. The amplified cDNA products of the *CB*_*1*_ (66 bp) and *CB*_*2*_ (141 bp) genes were detected by agarose gel electrophoresis (Table 1). Figure 2a and b shows the mRNA expression levels for *CB*_*1*_, *CB*_*2*_ and *PPIA* in RCC and ASE-5063 cells.

**Table 1:** Primer sequences used for *CB*_*1*,_ *CB*_*2*_ and *PPIA* genes

Gene	Primer sequences	Length (bp)
*CB*_*1*_	Forward primer: 5’-CGCTTTCCGGAGCATGTT-3’ Reverse primer: 5’-TCCCCCATGCTGTTATCCA-3’	66
*CB*_*2*_	Forward primer: 5’-TATGGGCATGTTCTCTGGAA-3’ Reverse primer: 5’-GAGGAGCACAGCCAACACTA-3’	141
*PPIA*	Forward primer: 5’-TGTGTCAGGGTGGTGACTTC-3’ Reverse primer: 5’-TTGCCATGGACAAGATGCCA-3’	123

### Expression of the cannabinoid receptor CB_2_ in RCC cells

**Fig. 3:**
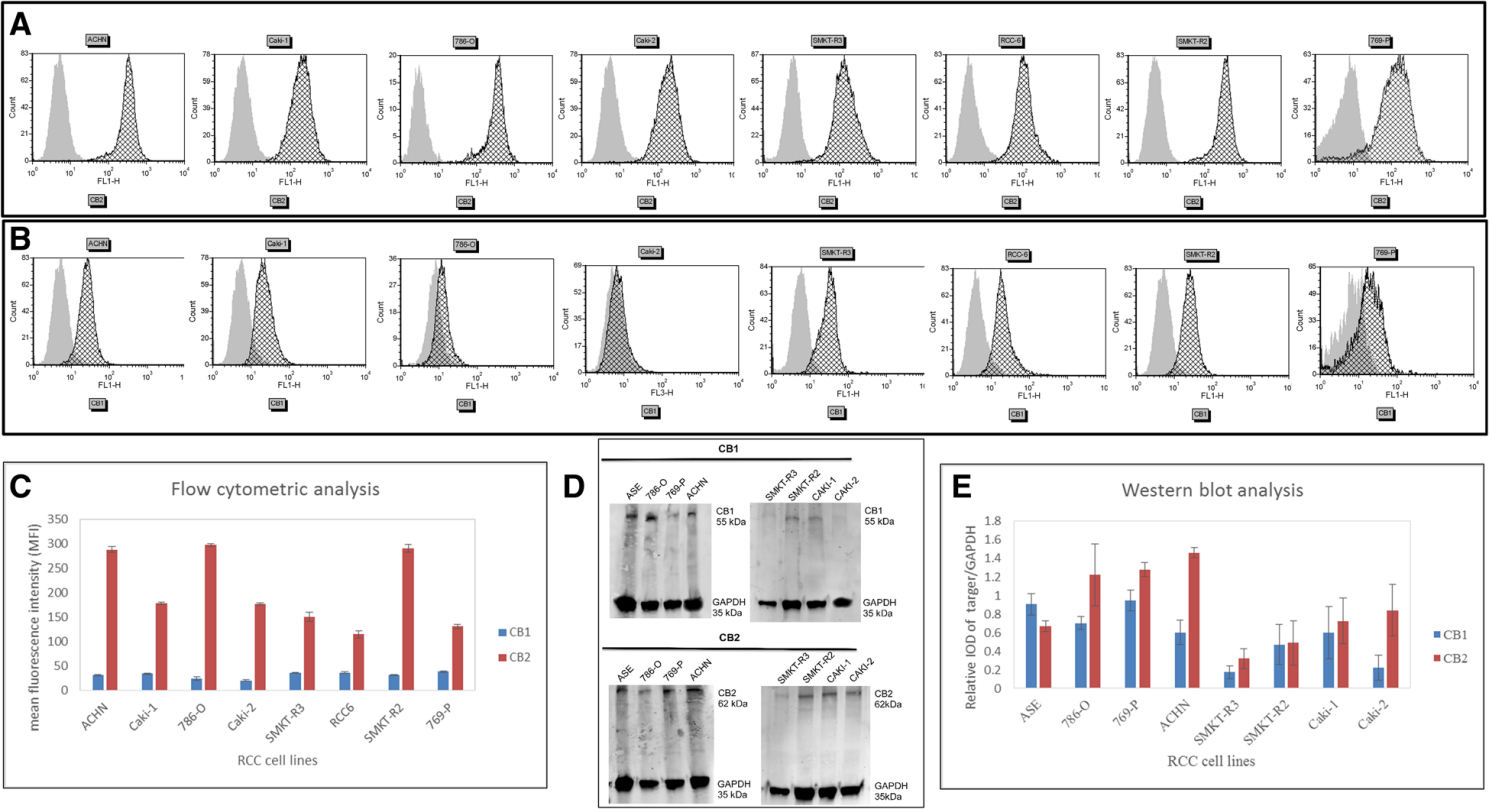
Flow cytometric and western immunoblot analysis of the CB_1_ and CB_2_ receptor proteins in RCC cells. Graphs showing the representative histograms of CB_2_-positive **a** and CB_1_-positive **b** cells from different RCC cell lines. In each of the RCC cell lines, the CB_2_ protein expression was higher than that of the CB_1_ protein. Gray-filled histogram, unstained cells; black line histogram, stained cells. **c** Quantitative data indicating the protein expression levels of the CB_1_ and CB_2_ receptors in RCC cells. **d** Western immunoblot of the CB_1_ and CB_2_ proteins in RCC cell lines. Healthy kidney ASE-5063 (ASE) cells were used as the positive control for the CB_1_ and CB_2_ proteins. The GAPDH protein was used as an internal control. Forty micrograms of total protein was loaded onto the gels in each case. In each lane, there were two bands of proteins, the top band for CB_1_ or CB_2_ and the lower band for GAPDH. **e** Quantitative analysis of the western blot

We used flow cytometry to analyze the expression of the membrane receptor proteins CB_1_ and CB_2_ in 8 different RCC cell lines. The objective of this experiment was to determine which of these proteins was highly expressed in RCC cells. Our flow cytometry analysis confirmed the expression of the CB_1_ and CB_2_ proteins in all the cell lines analyzed; however, more cells expressed the CB_2_ protein than the CB_1_ protein (Fig. 3a and b). Figure 3a and b displays representative histograms for the CB_1_ and CB_2_ protein expression, and the quantitative analysis of the CB_1_ and CB_2_ receptors in RCC cells is shown in Fig. 3c. The western blot analysis also revealed the protein expression of the CB_1_ and CB_2_ receptors in RCC cells. The receptors expressed in RCC cells had estimated molecular masses of approximately 55 kDa for CB_1_ and 62 kDa for CB_2_ (Fig. 3d and e). As a control for the CB_1_ and CB_2_ proteins, we used a protein lysate of healthy kidney ASE-5063 cells. GAPDH (35 kDa) was used as an internal control. Two immunoreactive bands were observed in each lane—one band corresponded to the cannabinoid receptor (CB_1_ or CB_2_) and the other band corresponded to GAPDH. The ICC results also corroborated these findings. The bands for the CB_1_ and CB_2_ proteins were observed to be somewhat higher than those corresponding to the 55-kDa and 62-kDa protein ladder markers, respectively, reflecting the glycosylated forms of the receptors.

### Immunocytochemical staining of the CB_2_ receptor

**Fig. 4:**
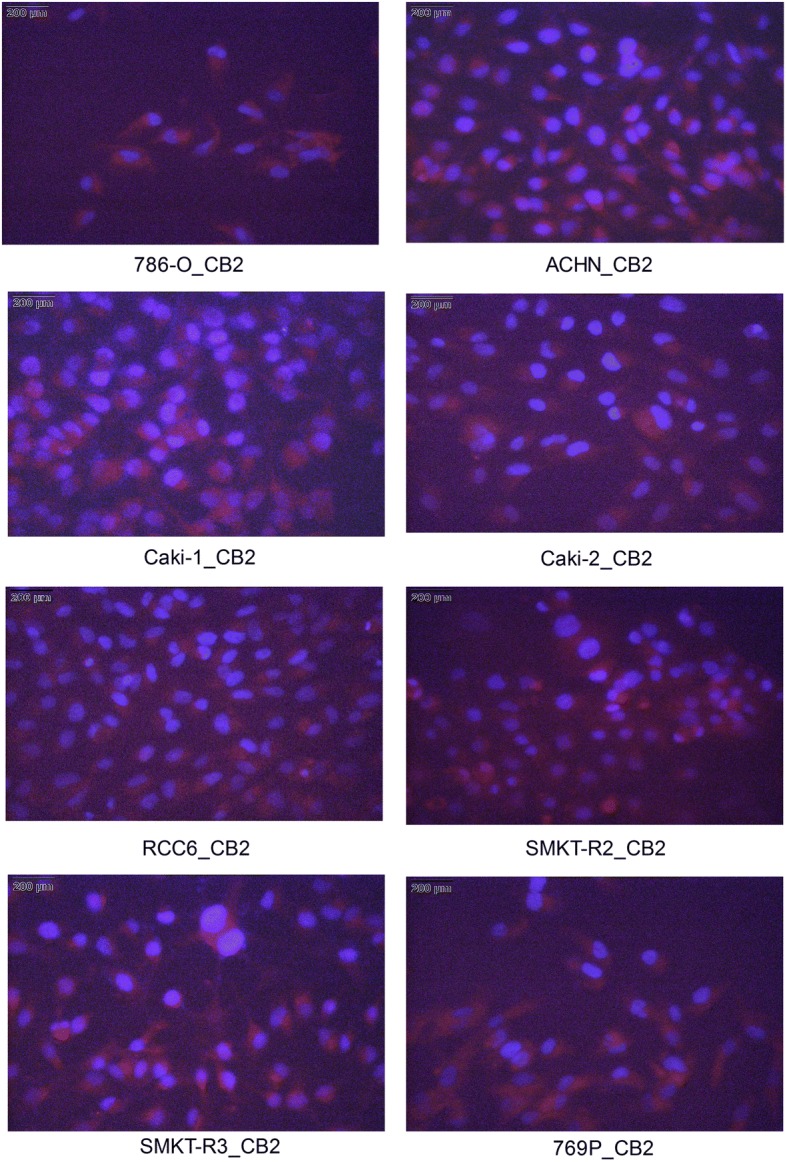
Immunocytochemical (ICC) staining of cannabinoid receptors. ICC was used to stain the CB_2_ receptor; CB_2_ was detected in fixed RCC cell lines. The cells were stained with anti-CB_2_ antibody and Alexa Fluor® 546 secondary antibody (red) and were counterstained with the nuclear dye DAPI (blue)

Immunocytochemical (ICC) is a highly productive method in biomedical research. We used this method to further localize the expression of the CB_1_ and CB_2_ proteins in RCC cells. Our results indicate that RCC cells expressed the CB_2_ protein (Fig. 4), while the CB_1_ protein was weakly expressed in these cells (data not shown). This finding is consistent with results obtained by flow cytometric and western blot analyses, in which low levels of CB_1_ protein expression were observed compared with the CB_2_ protein. For the control, RCC cells were separately stained with only the Alexa Fluor® 546 secondary antibody to determine whether the labeling was specific to the primary antibody.

### The cannabinoid WIN-55 inhibited the growth of RCC cells

**Fig. 5:**
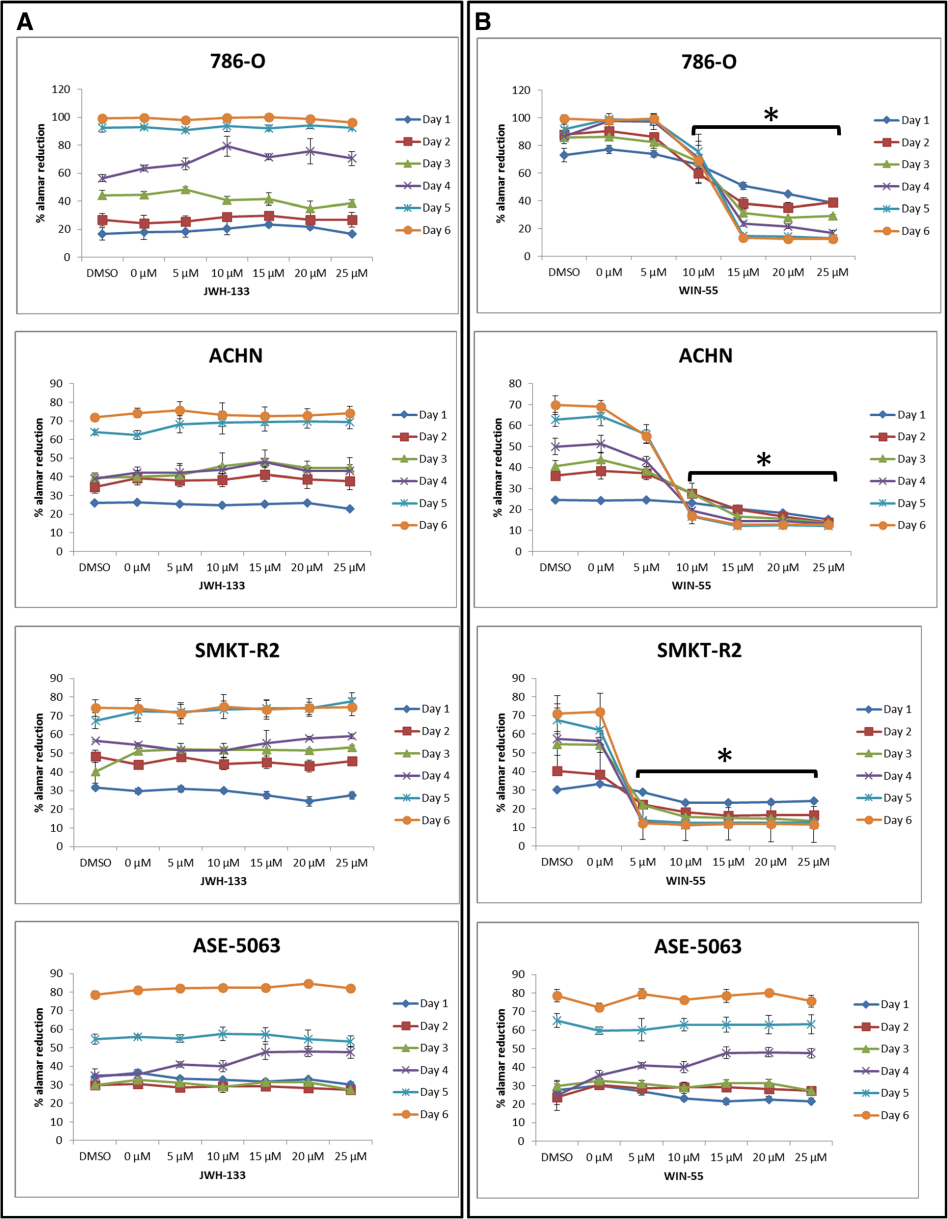
Percentage of reduction in cell viability according to the Alamar Blue® assay in RCC cell lines treated with JWH-133 and WIN-55. Representative graphs showing the cannabinoid effect on RCC cells and healthy kidney epithelial cells (ASE-5063). All of the cell lines were treated with increasing concentrations (0–25 μM) of JWH-133 (**a**) or WIN-55 (**b**), and cell proliferation was measured using Alamar blue reduction for 6 days. The agonist WIN-55 reduced the proliferation of the RCC cells, while JWH-133 did not produce a similar result [* *p* < 0.05 vs control (0 μM or DMSO)]

In these experiments, we first examined the anti-proliferative effects of the two synthetic cannabinoid agonists JWH-133 and WIN-55 on the RCC cell lines. JWH-133 is a highly selective CB_2_ receptor agonist, while WIN-55 is a non-selective cannabinoid CB_1_ and CB_2_ receptor agonist. WIN-55 and JWH-133 were dissolved in DMSO, and the final concentration of DMSO was 0.1% (*v*/v). The kinetics of JWH-133- and WIN-55-induced cell death was observed for 6 days (Fig. 5), and cell death caused by WIN-55 was evident from day 2. RCC cells were incubated with increasing concentrations (0 μM, 5 μM, 10 μM, 15 μM, 20 μM, 25 μM) of JWH-133 or WIN-55, and cell proliferation was measured for 6 days using the Alamar Blue® cell proliferation assay. The control cells were treated only with DMSO or complete media (0 μM). The agonist WIN-55 reduced the proliferation of RCC cells in a dose-dependent manner, and the effects were apparent compared with the control from 10 μM to higher concentrations; moreover, the results were statistically significant. In contrast, JWH-133 did not produce similar results in the RCC cells. Furthermore, we used healthy human kidney epithelial cells (ASE-5063) treated with JWH-133 and WIN-55 to determine whether these agonists could also produce an anti-proliferative effect in healthy cells. Our results demonstrated that the cannabinoid receptor agonist WIN-55 is highly selective in exerting anti-proliferative effects only on RCC cells; healthy kidney cells were not affected.

### Role of the CB_2_ receptor in the growth inhibition of RCC cells

**Fig. 6:**
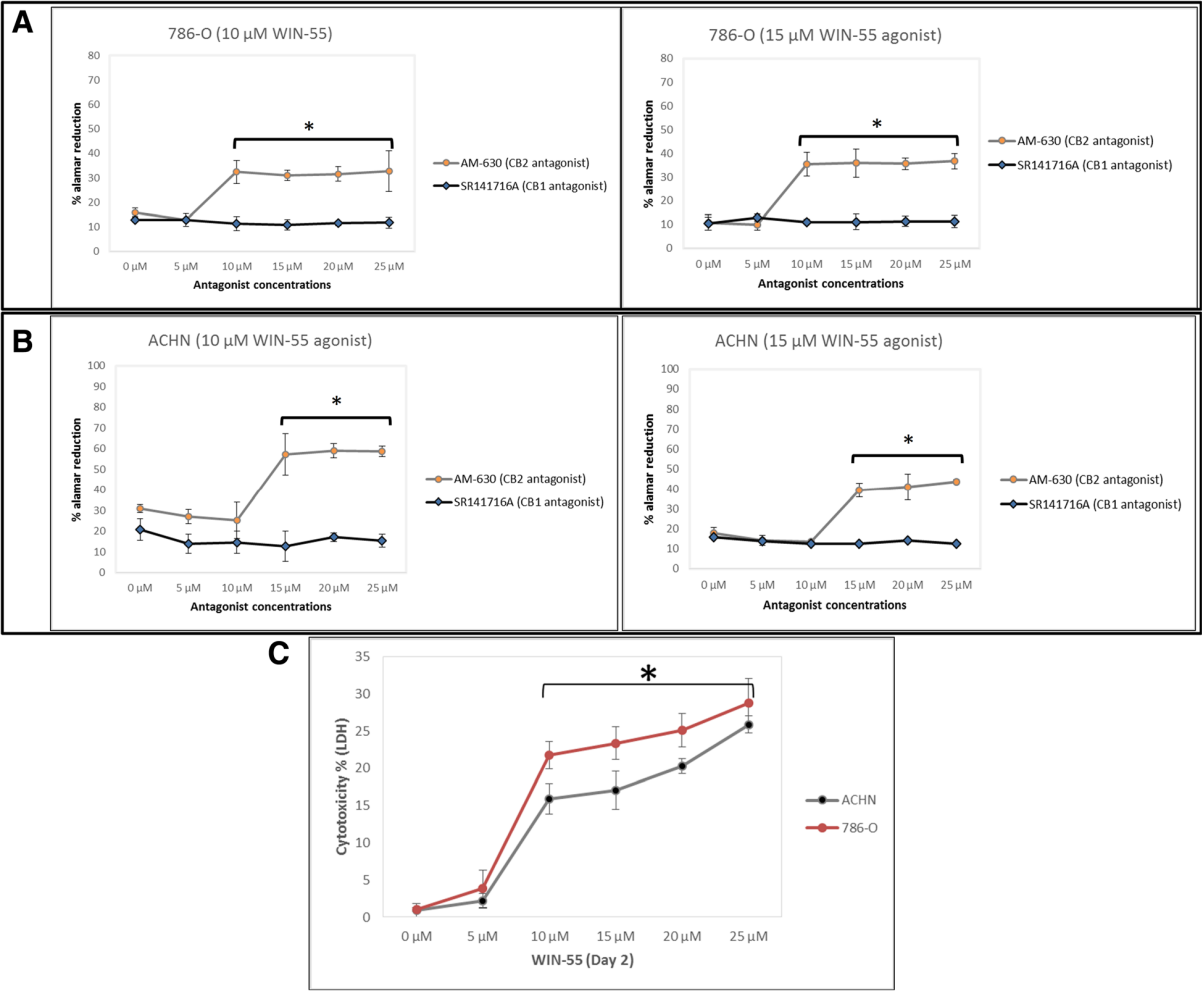
Inhibition of the cannabinoid-induced anti-proliferative effect by the CB_2_ antagonist AM-630. RCC cells (786-O (**a**) and ACHN (**b**)) were pre-treated with the concentration (0–25 μM) of antagonist AM-630 or SR141716A for 48 h before treatment with the agonist WIN-55 (10–15 μM). Representative graphs showing that blocking the CB_1_ receptor with the antagonist SR141716A and treatment with WIN-55 resulted in reduced RCC cell proliferation. However, cells pre-treated with the CB_2_ receptor antagonist AM-630 followed by WIN-55 treatment did not produce similar results, suggesting the involvement of CB_2_ in RCC cell proliferation [* *p* < 0.05 vs control (0 μM)]. **c** Cytotoxicity percentage of cultured RCC cells resulting from WIN-55 treatment. Graph showing RCC cells (786-O and ACHN) cultured with increase concentration of WIN-55 resulted in increased cytotoxicity based on lactate dehydrogenase (LDH) level. The cytotoxicity percentage was measured of release of LDH using Pierce LDH Cytotoxicity Assay Kit [* *p* < 0.05 vs control (0 μM)]

As shown in this study, RCC cells express both CB_1_ and CB_2_ receptors. Since WIN-55 is a non-selective cannabinoid receptor agonist for CB_1_ and CB_2_, we began to explore which cannabinoid receptor was responsible for the anti-proliferative action in RCC cells. Therefore, we pharmacologically blocked cannabinoid receptors separately with the CB_1_ receptor antagonist SR141716A and the CB_2_ receptor antagonist AM-630 in different experiments. After 48 h of antagonist treatment, the RCC cells were treated again with the agonist WIN-55, and proliferation was measured for 6 days by the Alamar Blue® cell proliferation assay. When blocking CB_2_ with its antagonist AM-630, the proliferation rate was not reduced during treatment of the RCC cells with the agonist WIN-55. In contrast, blocking CB_1_ with its antagonist SR141716A followed by treatment with the agonist WIN-55 reduced the proliferation of the RCC cells (Fig. 6a and b). These results suggest that CB_2_ is involved in the anti-proliferative action against RCC cells. We confirmed this result, which showed that the selectivity of the agonist WIN-55 for the CB_2_ receptor results in an anti-proliferative action in RCC. As a control, we also treated RCC cells with the antagonist SR141716A alone to determine whether the antagonist had any anti-proliferative effect.

### WIN-55 produces cytotoxic effect on RCC cells

We further evaluated RCC cells death caused by treatment with agonist WIN-55 using lactate dehydrogenase (LDH) release into the incubation medium. The LDH release graph for 786-O and ACHN cell lines treated with different concentrations of WIN-55 (0–25 μM) suggested that the cytotoxic effect of the WIN-55 was concentration-dependent (Fig. 6c). The percentage of LDH release from 786-O and ACHN cells treated with 10 μM, 15 μM, 20 μM, 25 μM of WIN-55 were 21, 23, 25, 28, and 15%, 17, 20, 25%, respectively, after 48 h of treatment. The cytotoxic effect was greater in 786-O cells in comparison to ACHN cells.

### WIN-55 inhibits proliferation of RCC cells to form 3D spheres/spheroids

**Fig. 7:**
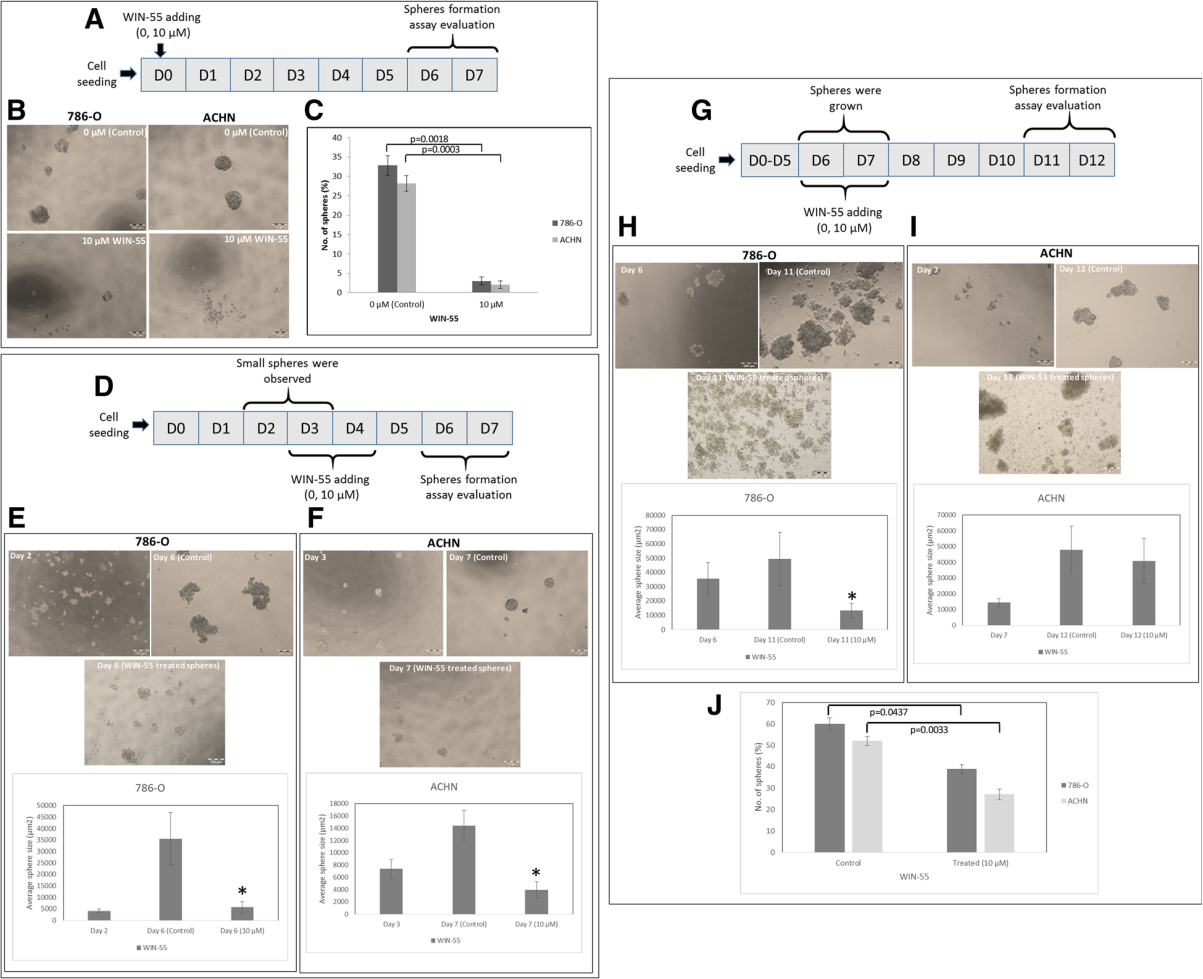
WIN-55 inhibits the proliferation of RCC cells into 3D spheres. Scheme of experiment design (**a**) (**d**) and (**g**)**. b** Representative images of spheres formed by 786-O and ACHN cells without/with WIN-55 (0, 10 μM). RCC cells were not able to form spheres (day 6–7) when WIN-55 was added at the beginning (day 0) of the assay. **c** Quantitative analysis of RCC spheres from 786-O and ACHN cell lines treated with WIN-55 (0, 10 μM). **d** Representative images and quantitative analysis of spheres formed by RCC cells when WIN-55 was added at day 2 for 786-O cells (**e**) and day 3 for ACHN cells (**f**). The growth of the spheres was observed at day 6 (786-O) and day 7 (ACHN) for quantitative analysis. **g** Representative images and quantitative analysis of sphere formation when WIN-55 was added at day 6 (786-O) (**h**) and day 7 (ACHN) (**i**). The growth of the spheres was observed at day 11 (786-O) and day 12 (ACHN) for quantitative analysis. **j** Quantitative analysis of RCC spheres treated with WIN-55 (0, 10 μM) at day 11 (786-O) and day 12 (ACHN). D: day; scale bar 200 μm; * *p* < 0.05

Sphere forming ability of RCC cells was inhibited by WIN-55 treatment when drug was added at the beginning (day 0). In the presence of WIN-55, single RCC cells were not able to proliferate into spheres in comparison to non-treated cells (0 μM (control)) (Fig. 7(a-c)). In other experiments, we added WIN-55 in the culture media when RCC cells started to form small spheres/spheroids (day 2–3) (Fig. 7 (d-f)). In this condition WIN-55 inhibited RCC cells proliferation into bigger spheres and changed spheres morphology. Proliferation of RCC spheres was halted and size of spheres was reduced. No further growth was observed and cells were loosely attached in culture.

In addition, we study the effect on sphere formation when WIN-55 was added after a longer period of growth (Fig. 7g). Figure 7h shows that the ability of 786-O cells to achieve spheres was reduced in form of size and number of spheres (Fig. 7j). Figure 7i shows ACHN cells were able to form similar size spheres but in reduced number (Fig. 7j). In both cell lines more floating, loosely attached cells were observed in culture media in comparison to control.

### WIN-55 induced G0/G1 arrest and apoptosis in RCC cells

**Fig. 8:**
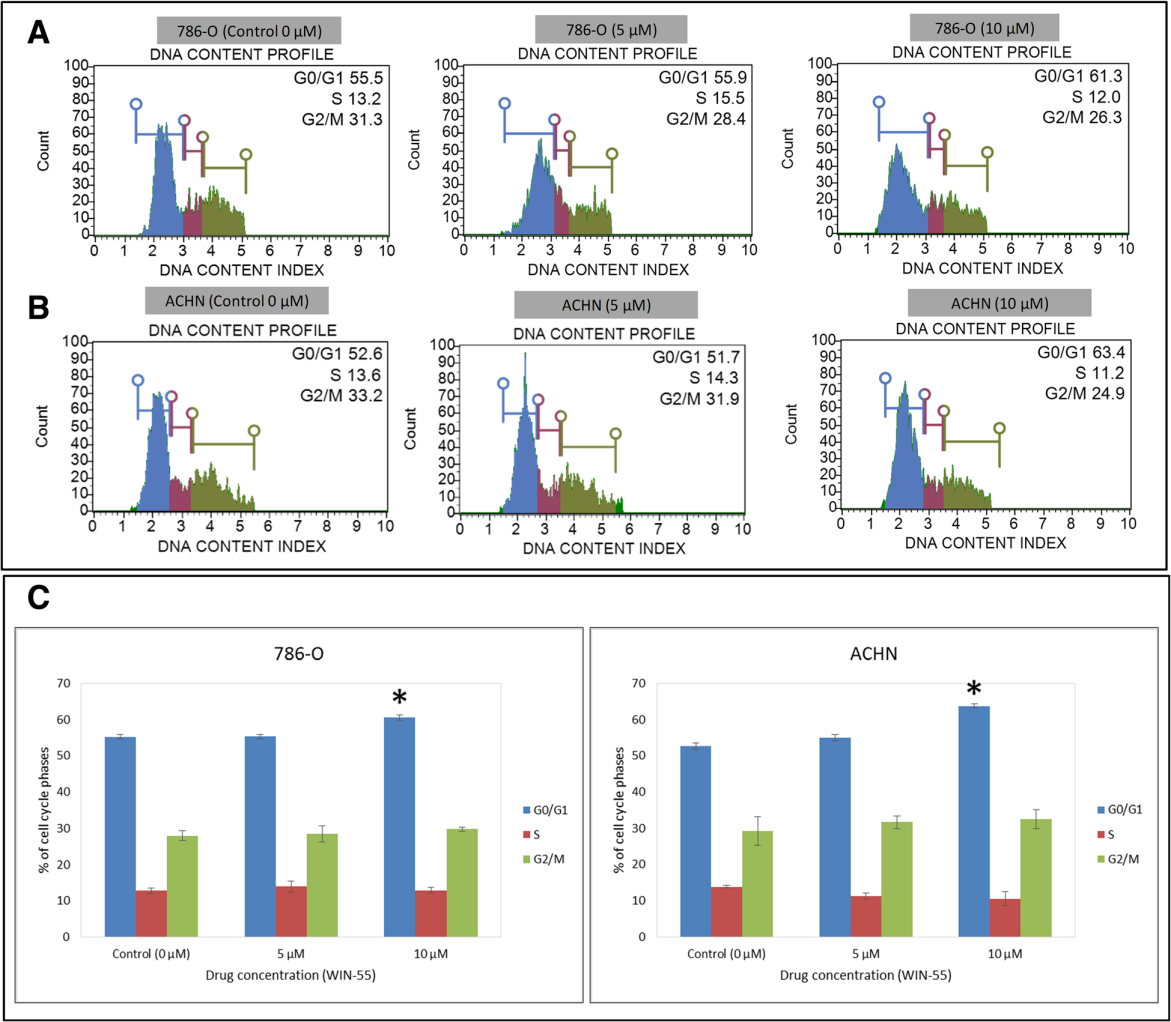
Cell cycle analysis of RCC cells. 786-O (**a**) and ACHN (**b**) cells were treated with increasing concentrations of WIN-55 for 48 h and analyzed using Muse™ Cell analyzer. **a** Representative cell cycle plots for the 786-O and **b** ACHN cell lines at different drug concentrations (0 μM, 5 μM and 10 μM). **c** Quantitative data indicating a significant arrest of WIN-55-treated (10 μM) 786-O (61%) and ACHN (63%) cells in the G0/G1 cell cycle phases compared to control (0 μM) [* *p* < 0.05]

**Fig. 9:**
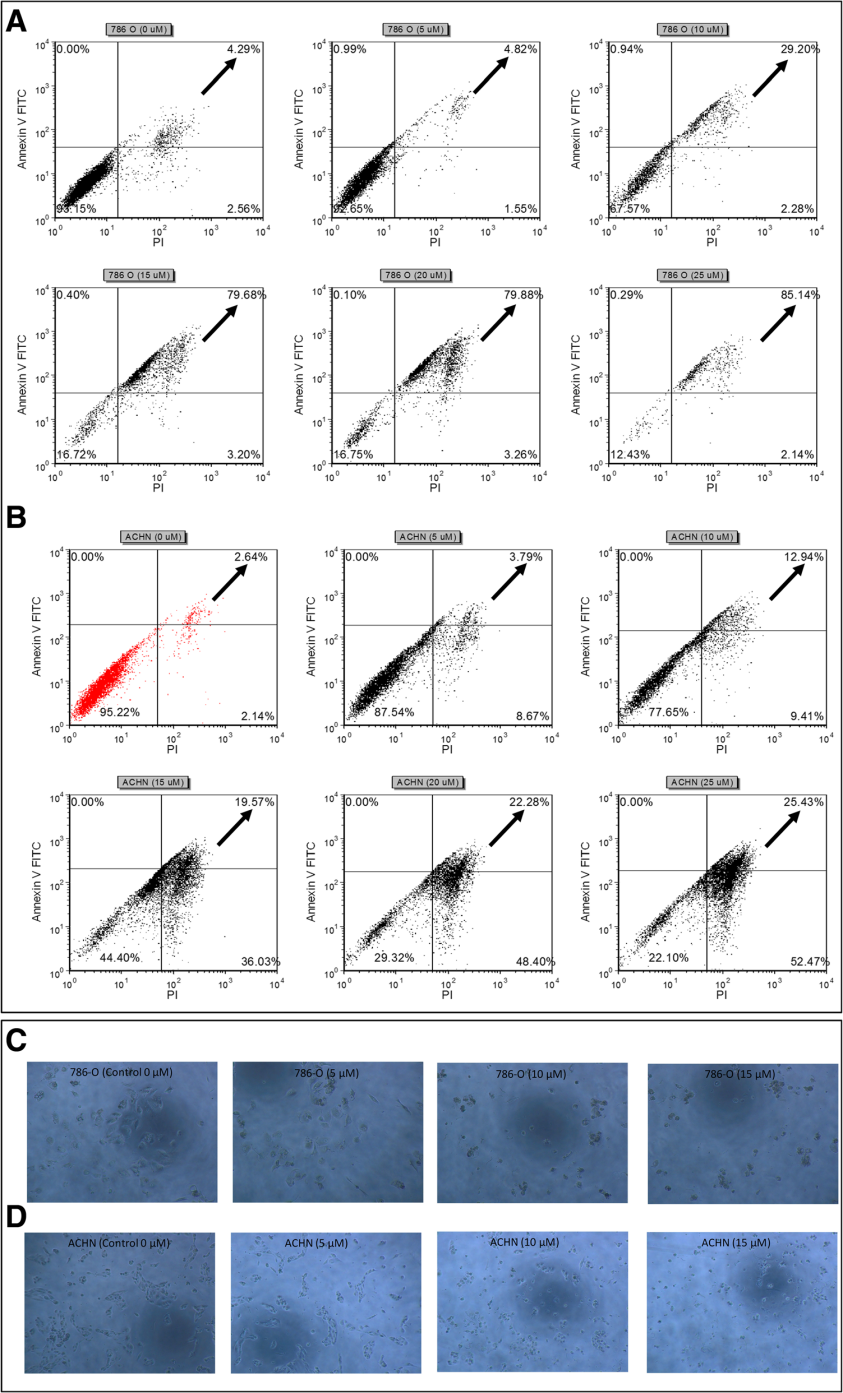
WIN-55 induced apoptosis analysis in RCC cells. WIN-55 induced apoptosis of 786-O (**a**) and ACHN (**b**) cells as assessed by flow cytometry using Annexin V-FITC and PI. Black arrows in each dot plot represents increase in late apoptotic cells with increased concentration of WIN-55. Morphological changes of 786-O (**c**) and ACHN (**d**) cells treated with increasing concentrations of WIN-55 for 48 h and examined under light microscopy [* *p* < 0.05 vs control (0 μM)]

To investigate the mechanisms responsible for the anti-proliferative effect of WIN-55, its effect on cell cycle progression of the 786-O (Fig. 8a) and ACHN (Fig. 8b) cells was determined by flow cytometry as a function of the agonist concentrations. The G0/G1 cell population was significantly changed at concentration of 10 μM in both cell lines (in 786-O cells from 55 to 61% and in ACHN cells 52 to 63%) compared with the control sample (0 μM) (Fig. 8c). These data indicated that WIN-55 effectively inhibited cell proliferation at concentrations higher than 10 μM, leading to apoptotic cell death in the RCC cells (Fig. 9). These results also indicated that WIN-55 induced a G0/G1 arrest, which might result in apoptosis in the 786-O (Fig. 9a) and ACHN (Fig. 9b) cells. In addition, microscopic examination also revealed morphological changes in cell shape, cell shrinking, gradient decrease in living cells, and an increased number of less adherent cells in the culture media (Fig. 9c and d).

### Effects of WIN-55 treatment on PI3K/MAPK expression of RCC cells

**Fig. 10:**
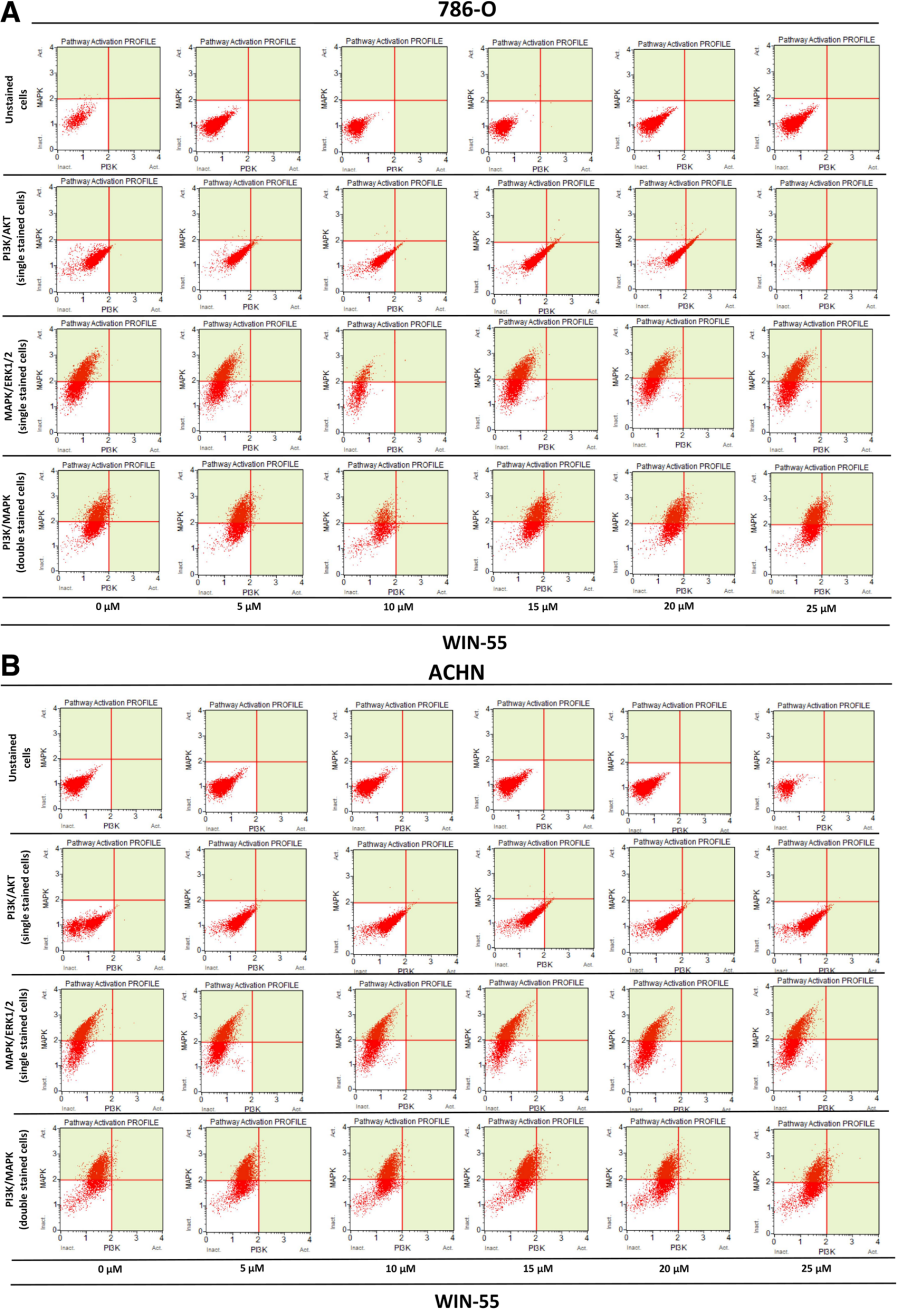
PI3K/Akt and MAPK/ERK1/2 pathways activation profile of RCC cells. **a** Dual pathway (PI3K and MAPK) profile of 786-O cells treated with agonist WIN-55 (0–25 μM). **b** Dual pathway (PI3K and MAPK) profile of ACHN cells treated with agonist WIN-55 (0–25 μM). ACHN and 786-O cells were found positive for Phospho-ERK1/2 (MAPK/ERK1/2) but negative for Phospho-Akt (PI3K/Akt). The dot plots showing WIN-55 treatment does not activate/inhibit PI3K and MAPK pathways even in higher concentrations (10–25 μM) as compared to the not treated control (0 μM, first column in Fig. **a** and **b**). In each fig. **a** and **b** dot blots in second and third raw represents cells single stained with Phospho-Akt (PI3K/Akt) and Phospho-ERK1/2 (MAPK/ERK1/2), respectively. Dot blots in fourth raw in each fig. (a and b) represents cells double stained with Phospho-Akt (PI3K/Akt) and Phospho-ERK1/2 (MAPK/ERK1/2)

In this study, we investigated the PI3K/Akt and MAPK/ERK1/2 pathways using flow cytometry assay in order to determine whether the anti-proliferative action of WIN-55 is mediated by Akt and/or ERK1/2 activation or inhibition. As shown in Fig. 10 WIN-55 treatment neither activated nor inhibited the PI3K/Akt and MAPK/ERK1/2 signaling pathways. 786-O and ACHN cells were found positive for Phospho-ERK1/2 and negative for Phospho-Akt. Overall, these results did not show any involvement of WIN-55 mediated activation or inhibition of PI3K/Akt and MAPK/ERK1/2 pathways.

## Discussion

**Fig. 11:**
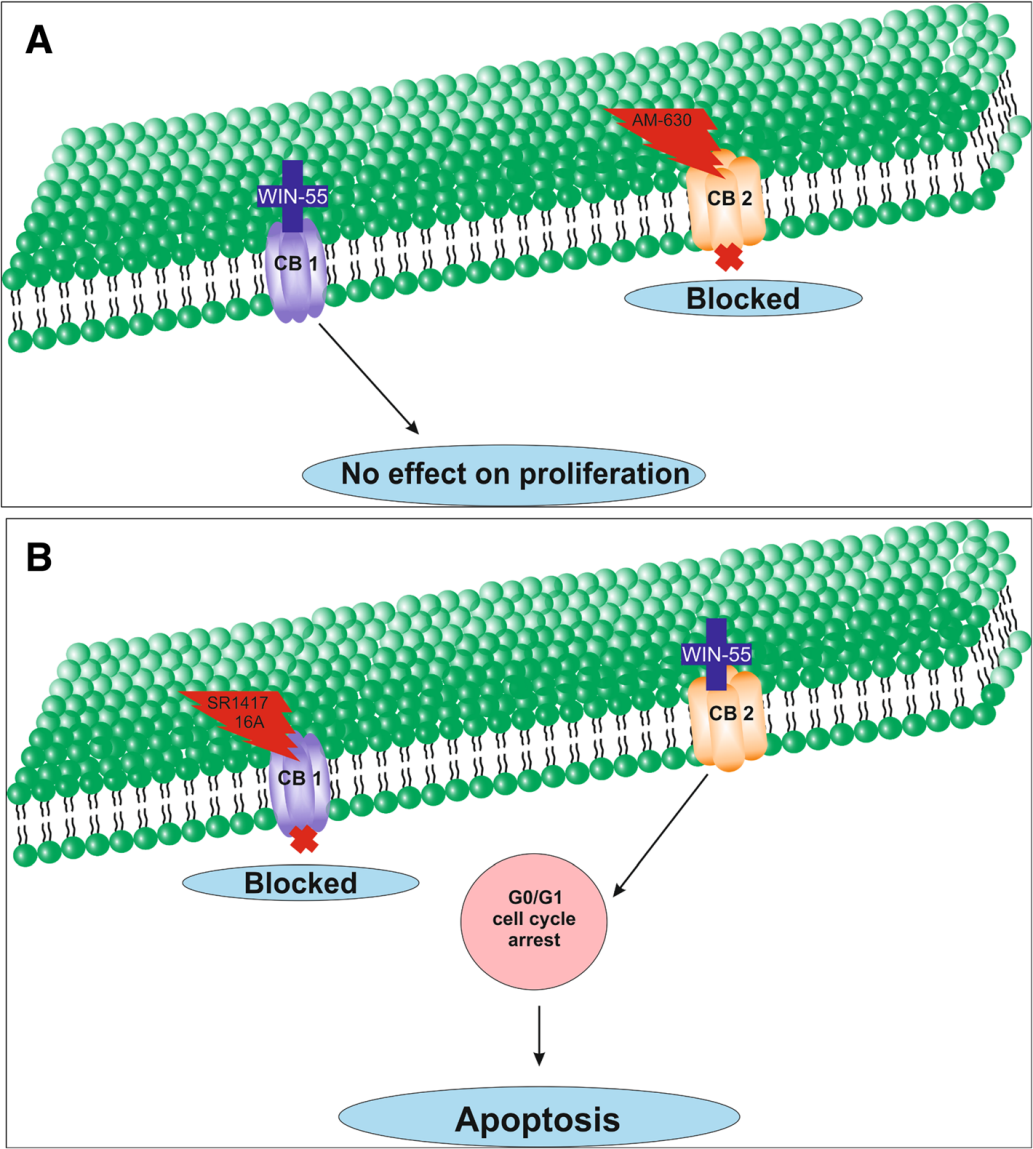
Schematic model of the apoptotic effect of WIN-55 on RCC cells. **a** WIN-55 binds to CB_1_ receptor in the presence of CB_2_ receptor antagonist (AM-630) have no effect on proliferation. **b** WIN-55 binds to the CB_2_ receptor in the presence of CB_1_ receptor antagonist (SR141716A) and induces G0/G1 arrest, which further leads to anti-proliferation and apoptotic cell death

The activation of cannabinoid receptors (CB_1_ and CB_2_), which comprise the ECS, participates in several physiological processes inside cells [12]. There is a growing number of studies that shed light on the use of the ECS and cannabinoids to target cancer cells [10, 15, 17, 29]. To investigate the anti-cancer effects and anti-cancer mechanisms of cannabinoids on RCC cells, we first explored the mRNA and protein expression of CB_1_ and CB_2_ receptors on established RCC cell lines. In this study, we found that both the CB_1_ and CB_2_ receptors were expressed in 8 different RCC cell lines. However, the expression of CB_2_ was higher than that of CB_1_ within the same cell line. These data were also confirmed by real-time PCR assays, flow cytometry, western blot analysis and ICC (Figs. 2, 3 and 4). Others have also analyzed the expression of cannabinoid receptors in RCC tissue and surrounding non-neoplastic kidney tissue [30]. The expression of the CB_1_ receptor was shown only at the mRNA level, while the protein expression of CB_1_ was absent. Moreover, CB_2_ expression was not detectable at either the mRNA or protein level in tumor and normal tissue. Therefore, our data suggest that CB_1_ and CB_2_ receptors could be novel targets for RCC treatment options. We also demonstrated that the synthetic cannabinoid WIN-55, a non-selective cannabinoid CB_1_ and CB_2_ receptor agonist, inhibits the proliferation of RCC primary (786-O) and metastatic (ACHN) cells through the activation of the CB_2_ receptor as determined by the Alamar Blue® cell viability assay. WIN-55 treatment of 786-O and ACHN cells resulted in a decrease in cell viability with various drug concentrations starting at 10 μM, and the effects were visible after 48 h of treatment. In the SMKT-R2 cell line, the anti-proliferative effects were visible from 5 μM WIN-55. We also analyzed whether these synthetic cannabinoids produced similar effects on non-cancer cells. For this purpose, we used the healthy kidney epithelial cell line (ASE-5063). Our observation confirmed that the cannabinoids produced anti-proliferative effects only on cancer cells, while non-cancer cells such as ASE-5063 cells avoided these effects (Fig. 5). This apparent selectivity of cannabinoids (WIN-55 and JWH-133) for cancer cells makes the ECS together with the CB_1_ and CB_2_ receptors an attractive target for cancer prevention. However, it remains unknown how cannabinoids distinguish cancer cells from non-cancer cells, and further research is required to gain this knowledge. Apoptosis is an ideal approach to eliminating cancer cells, and the selective killing of cancer cells by apoptosis could provide a better understanding of the proper elimination of cancer cells and cancer prevention. In this study, we also observed an increase in apoptotic cells in the 786-O and ACHN cell lines with WIN-55 treatment. These results were confirmed by flow cytometry, LDH-based cytotoxic assay, in vitro sphere formation assay and morphological changes were observed by light microscopy (Figs. 6c, 7 and 9). WIN-55 was able to inhibit in vitro proliferation of RCC cells into 3D spheres (Fig. 7). WIN-55, which is a mixed CB_1_/CB_2_ agonist, produced anti-proliferative effects in RCC cells, raising another question: which cannabinoid receptor (CB_1_ or CB_2_) was involved in the anti-proliferative action in RCC cells? We demonstrated the involvement of the CB_2_ receptor in the anti-proliferative effect in RCC cells by pharmacologically blocking the CB_1_ and CB_2_ receptors separately using the CB_1_ receptor antagonist SR141716A and the CB_2_ receptor antagonist AM-630 (Fig. 6a and b). These results confirmed the data observed in different cancers, which also revealed that the CB_2_ receptor is involved in the proliferation, differentiation and survival of cancer cells [15, 31, 32]. Our observation is consistent with the level of CB_2_ expression in RCC cells, and CB_2_ receptor stimulation by WIN-55 could be involved in the anti-tumor activity. There are studies that have shown that apoptotic cell death may be due to arrest at a particular phase of the cell cycle [33–35]. Therefore, we performed a cell cycle analysis of 786-O and ACHN cells after treatment with WIN-55. We observed that treatment with WIN-55 caused arrest in the G0/G1 phase of the cell cycle in the RCC cells, which further led to apoptotic cell death (Fig. 8). The number of cells accumulating in the G0/G1 phase also increased with increasing doses of WIN-55. These findings correlate with the results observed in the human prostate cancer cell line LNCaP and human gastric cancer cell lines (AGS, MKN-1 and SNU-620), in which treatment with the agonist WIN-55 leads to arrest in the G0/G1 phase of the cell cycle [36–38]. There are many reports that have demonstrated that PI3K/Akt and MAPK/ERK signaling pathways are involved in the control of cell proliferation and cell survival in different cancer type of cells when treated by cannabinoid agonists [39–42]. Moreover, WIN-55 was reported to play an important functional role through activating or enhancing the PI3K/Akt pathway, induces cell cycle arrest, inhibits the proliferation and migration of human hepatocellular carcinoma cells [40]. WIN-55 was also reported to inhibit differentiation of prostate cancer cells via down regulating the PI3K/Akt/mTOR signaling pathway [42]. In order to determine anti-proliferative mechanism of WIN-55 in RCC cells, we analyzed the activation of Akt and/or ERK1/2 after RCC cells were treated with WIN-55 and analyzed using flow cytometry. Our results show that WIN-55 treatment does not activate nor inhibit PI3K/Akt and MAPK/ERK1/2 signaling pathways in RCC cells (Fig. 10). These results suggest that other signaling pathways might be involved in the anti-proliferative action of WIN-55 treatment. Overall, with the anti-tumor action of cannabinoids against RCC in vitro, this study showed the therapeutic potential of the cannabinoid receptor CB_2_ (Fig. 11). There is no doubt that an improved understanding of the pathways and downstream mechanisms induced by cannabinoids in RCC is also necessary, and this knowledge could be useful in combination with other therapies for better management and prevention of RCC. Moreover, CB_2_ receptor agonists do not cause the central nervous system effects typically produced by cannabinoid ligands with agonist activity at the CB_1_ receptor [13, 43], a characteristic that could be exploited for future cannabinoid-based anti-cancer therapies.

## Conclusions

In summary, our study shows the involvement of CB_2_ receptor in the in vitro inhibition of RCC cells. This knowledge will be useful to unravel the future applications of CB_2_ receptor and its agonists in the prevention and management of RCC.

## Abbreviations

Akt: Protein kinase B
AM-630: 6-iodo-2-methyl-1-[2-(4-morpholinyl)ethyl]-1H-indol-3-yl](4-methoxyphenyl)methanone
CB_1_: Cannabinoid receptor type 1
CB_2_: Cannabinoid receptor type 2
DMSO: Dimethyl sulfoxide
ECS: Endocannabinoid system
FBS: Fetal bovine serum
FcR: Fc receptor
ICC: Immunocytochemistry
IFNα: Interferon-alpha
IOD: Integrated optical density
JWH-133: (6aR,10aR)-3-(1,1-dimethylbutyl)-6a,7,10,10a-tetrahydro-6,6,9-trimethyl-6H-dibenzo[b,d]pyran
LDH: Lactate dehydrogenase
mTOR: mammalian target of rapamycin
PBS: Phosphate-buffered saline
PD-1: Programmed death-1
PFA: Paraformaldehyde
PI3K: Phosphoinositide 3-kinase
RCC: Renal cell carcinoma
SR141716A: N-(piperidin-1-yl)-5-(4-chlorophenyl)-1-(2,4-dichlorophenyl)-4-methyl-1H-pyrazole-3-carboxamide hydrochloride
TKIs: Tyrosine kinase inhibitors
VEGF: Vascular endothelial growth factor
WIN 55,212–2 (WIN-55): (R)-(+)-[2,3-dihydro-5-methyl-3-(4-morpholinylmethyl)pyrrolo[1,2,3-de]-1,4-benzoxazin-6-yl]-1-naphthalenylmethanone mesylate
MAPK: Mitogen-activated protein kinase
ERK: Extracellular signal-regulated kinase
